# Enhanced healing efficacy of an optimized gabapentin-melittin nanoconjugate gel-loaded formulation in excised wounds of diabetic rats

**DOI:** 10.1080/10717544.2022.2086943

**Published:** 2022-06-24

**Authors:** Hani Z. Asfour, Nabil A. Alhakamy, Osama A. A. Ahmed, Usama A. Fahmy, Shadab Md, Mohamed A. El-Moselhy, Waleed Y. Rizg, Adel F. Alghaith, Basma G. Eid, Ashraf B. Abdel-Naim

**Affiliations:** a Department of Medical Microbiology and Parasitology, Faculty of Medicine, King Abdulaziz University, Jeddah, Saudi Arabia; b Department of Pharmaceutics, Faculty of Pharmacy, King Abdulaziz University, Jeddah, Saudi Arabia; c Center of Excellence for Drug Research and Pharmaceutical Industries, King Abdulaziz University, Jeddah, Saudi Arabia; d Mohamed Saeed Tamer Chair for Pharmaceutical Industries, King Abdulaziz University, Jeddah, Saudi Arabia; e Department of Clinical Pharmacy and Pharmacology, Ibn Sina National College for Medical Studies, Jeddah, Saudi Arabia; f Department of Pharmaceutics, College of Pharmacy, King Saud University, Riyadh, Saudi Arabia; g Department of Pharmacology and Toxicology, Faculty of Pharmacy, King Abdulaziz University, Jeddah, Saudi Arabia

**Keywords:** Diabetes, wound healing, gabapentin, melittin, nanoconjugation

## Abstract

The present study aimed to design and optimize, a nanoconjugate of gabapentin (GPN)-melittin (MLT) and to evaluate its healing activity in rat diabetic wounds. To explore the wound healing potency of GPN-MLT nanoconjugate, an in vivo study was carried out. Diabetic rats were subjected to excision wounds and received daily topical treatment with conventional formulations of GPN, MLT, GPN-MLT nanoconjugate and a marketed formula. The outcome of the in vivo study showed an expedited wound contraction in GPN-MLT-treated animals. This was confirmed histologically. The nanoconjugate formula exhibited antioxidant activities as evidenced by preventing malondialdehyde (MDA) accumulation and superoxide dismutase (SOD) and glutathione peroxidase (GPx) enzymatic exhaustion. Further, the nanoconjugate showed superior anti-inflammatory activity as it inhibited the expression of interleukin-6 (IL-6) and tumor necrosis factor-α (TNF-α). This is in addition to enhancement of proliferation as indicated by increased expression of transforming growth factor-β (TGF- β), vascular endothelial growth factor-A (VEGF-A) and platelet-derived growth factor receptor-β (PDGFRB). Also, nanoconjugate enhanced hydroxyproline concentration and mRNA expression of collagen type 1 alpha 1 (Col 1A1). In conclusion, a GPN-MLT nanoconjugate was optimized with respect to particle size. Analysis of pharmacokinetic attributes showed the mean particle size of optimized nanoconjugate as 156.9 nm. The nanoconjugate exhibited potent wound healing activities in diabetic rats. This, at least partly, involve enhanced antioxidant, anti-inflammatory, proliferative and pro-collagen activities. This may help to develop novel formulae that could accelerate wound healing in diabetes.

## 1. Introduction

The defensive role of the dermal layer as a first-line in exhibiting protection against pathogens or against physical injury is well established (Strecker-McGraw et al., 2007). A healthy and integrated dermal layer performs the crucial role of maintaining check and balance in the human body. When minor wear-tear occurs, the skin automatically heals itself, and the damaged layer sheds off (Tottoli et al., 2020). However, when skin integrity gets compromised in response to multiple traumas, coexisting disease conditions such as diabetes, or in a stressed condition, the healing capacity gets significantly affected, and chronic cutaneous wounds persist (Qing, 2017). The ideal definition of dermal/cutaneous wound refers to the loss of anatomical and functional integrity of the epidermis, dermis and endodermis. In a normal physiological condition, the cutaneous wound healing begins immediately and may last for months, depending on multiple factors (Li et al., 2022a). The cost of wound treatment has been expected to increase significantly over the next decade because of diabetes and the modern lifestyle (Velnar et al., 2009). Nevertheless, the wound healing mechanism is a dynamic and complex process that involves multiple factors such as the role of inflammation, oxidative stress, apoptosis, and fibrosis (Roy et al., 2022). The precise wound healing mechanism comprises a role of growth factors such as transforming growth factor-beta (TGF-β), cytokines such as transforming growth factor beta (TNF-α), interleukins (ILs), angiogenic factors such as vascular endothelial growth factor (VEGF) and fibrotic factors (Wan et al., 2021).

Among various types of wounds, the management and treatment of diabetic wounds have remained an area of concern for health care professionals (Deng et al., 2021). Based on numerous clinical and preclinical evidence, connecting link between hyperglycemia and impaired wound healing has been well established (Patel et al., 2019). It is well established that normal wound healing is a controlled anticipated process, and any alteration in the steps of the healing process may result in “venous ulcer,” ‘intractable ulcers or chronic wounds that sometimes become lethal. Hyperglycemic condition hinders the delivery of nutrients and other immunological factors that automatically reflect impaired wound healing (Jiang et al., 2022). In diabetic patients, delayed wound healing is related to impaired growth factor production, inflammatory cell function, angiogenesis and fibroblast production and migration (Rose & Kam, 2002). Therefore, novel and targeted pharmacological approaches are urgently needed to expedite the healing of diabetic wounds.

Gabapentin (GPN) is a major drug that is extensively used as an anticonvulsant and to treat neuropathic pain in diabetic patients (Kukkar et al., 2013). GPN is one of the structural analogs of gamma-aminobutyric acid and was approved for use in the nineties (Ola et al., 2019). GPN acts via the inhibition of the α (2)/δ subunit of voltage‐dependent calcium channels (Sarıtaş et al., 2016). Experimentally, GPN has been reported to exhibit antioxidant and antiapoptotic effects in the retina of diabetic rats (Gudeman et al., 2013). GPN has been to possess wound healing properties in rats (Gajski & Garaj-Vrhovac, 2013). Clinically, GPN may be compounded and used to promote dermal healing when conventional therapies fail or cannot be tolerated by the patient (Lee & Bae, 2016). Melittin (MLT) is a major peptide constituent of bee venom that has been reported to possess a plethora of medicinal uses (Carpena et al., 2020). It has been shown to exhibit anti-inflammatory (Jeong et al., 2015), antioxidant (Al-Ani et al., 2015), antiviral (Alhakamy et al., 2021b), antifungal (Memariani & Memariani, 2020), antimicrobial (Carpena et al., 2020) and pro-collagen (Gajski & Garaj-Vrhovac, 2013) properties. In addition, MLT has been shown to expedite wound healing in rats (Al-Ani et al., 2015; Carpena et al., 2020).

Wound nanotherapy offers a number of possibilities for overcoming current barriers in wound therapeutics. Nanostructures have a high surface-to-volume ratio, which has various benefits as drug carriers (Siddiqui et al., 2014). Nanoconjugate formulation can increase surface area, saturation solubility, and drug release rate, resulting in the needed drug concentration at the targeted spot. Many drugs are unable to cross the dermal barrier in their natural state, but this can be solved by using a nanoconjugate formulation (Alhakamy et al., 2021b). Thus, it is hypothesized that combining GPN with MLT in a nano formula would offer enhanced activities required to expedite wound healing in diabetes. Therefore, the present study aimed at optimizing GPN-MLT nanoconjugate formulation using experimental design-based software. Further, the efficacy of the optimized nanoconjugate to expedite healing was evaluated in experimentally induced diabetic wounds in rats.

## 2. Material and methods

### 2.1. Drugs and chemicals

GPN, MLT, hydroxypropyl-methyl cellulose (HPMC) and streptozotocin were obtained from (Sigma Aldrich, MO, USA). All other chemicals used in the study were of analytical grade.

### 2.3. Experimental design used for the development of nanoconjugate

The optimization of the GPN-MLT nanoconjugate was carried out using the Behnken design. The pH, incubation time (min), and sonication time (min) were selected as the independent variables. The particle size of prepared nanoconjugate formulations was chosen as the dependent variable of the design (Table 1). The minimum particle size value was the goal of the experimental design. Statgraphics software (Statgraphics Technologies, Inc., Warrenton, VA, USA) was utilized to generate and evaluate the experimental design (Siddiqui et al., 2014).

**Table 1.** Selected variables required and levels for the development of the experimental design.

	Factors	Levels	
**Independent variables**	**Low**	**High**	**Optimum**
A = pH	2.0	7.0	2.0
B = Incubation time (min)	10.0	50.0	10.03
C = Sonication time (min)	2.0	8.0	8.0
	**Dependent variables**	**Goal**	
	*R*_1_ = Particle size (nm)	Minimum	

### 2.4. Preparation of GPN-MLT nanoconjugate

As per the runs obtained from the Statgraphics software, i.e. 15, different GPN-MLT loaded nano complex were prepared. To prepare the GPN-MLT nanoconjugate, the quantity of GPN (0.05 µM) and MLT (0.01 µM) were mixed properly and dissolved in 20 ml of 0.01 M phosphate buffer solution of different pH with continuously stirring. Next, these mixtures were incubated and sonicated as per the runs obtained from the Statgraphics software.

### 2.5. Optimization of GPN-MLT nanoconjugate

The numerical technique was used to optimize the nanoconjugate. During numerical optimization, the software established the minimum values for the response, i.e. particle size as the goal. The software's recommended optimum formula was then fabricated, characterized, and evaluated.

### 2.6. Particle size determination

The Malvern Zeta-Sizer Nano-ZS-90 (Worcestershire, UK) was used to analyze the particle size of the prepared nanoconjugates of GPN-MLT. For the particle size analysis, the material was diluted 100 times with distilled water, uniformly mixed, and examined at 25 °C, with a 90° scattering angle.

### 2.7. Formulation of GPN-MLT nanoconjugate loaded hydrogel

Aqueous solutions of GPN, MLT and optimized GPN-MLT nanoconjugate were added separately to HPMC (1.5% w/v concentrations) hydrogel. The formed hydrogels of each component (GPN and MLT and GPN- MLT) were kept at 4 °C temperature before further use.

### 2.8. Experimental animals

Male Wistar rats (200–240 g) were obtained from the Animal Facility of the Faculty of Pharmacy, King Abdulaziz University (KAU), Saudi Arabia. Animal handling was approved by the Research Ethics Committee, Faculty of Pharmacy, KAU (Reference # PH-1443-28). Animals were kept under a 12-h dark-light cycle and under a temperature of 22 ± 2 °C and humidity of 40–60%.

### 2.9. Induction of diabetes

For the induction of diabetic wound, a freshly prepared streptozotocin in a citrate buffer (0.1 M, pH 4.5) at the dose of 50 mg/kg was administered i.p.14 days before the initiation of the study. Confirmation of diabetes was done after estimation of blood glucose and only the rats having blood glucose levels between 200 and 300 mg/100 mL were selected for the study (Alhakamy et al., 2021b).

### 2.10. Wounding by excision and treatment protocol

Diabetic rats were anesthetized by IP injections of ketamine and xylazine (100 mg/kg and 10 mg/kg, respectively). Then, the dorsal surface of the skin of each rat was properly sterilized using the povidone-iodine solution, and an incision (circulate, 1 cm in diameter) was made. The induced incision was washed with the sterile solution and further dried using pads (sterile). Furthermore, to reduce the pain due to incision, 2% lidocaine solution having ephedrine (4.4 mg/kg) in the ratio of 1:80,000 was administered subcutaneously. Once the diabetic wounds were induced, animals were divided into 6 groups (*n* = 6). Group 1 (untreated), only with induced wound and no treatment. Group 2 (vehicle), only treated with the vehicle (plain HPMC hydrogel). Group 3 (GPN), was treated with the hydrogel formulation of GPN whereas Group 4 (MLT), was treated with the hydrogel formulation of MLT. Group 5 and 6 (GPN-MLT and positive control), were treated with nanoconjugate of GPN-MLT loaded hydrogel and marketed formulation (Mebo®). Except, for untreated groups, various formulations were applied topically for 14 days to all the animals. Wounds in all the animals were covered with sterile pads and changed once a day. Wounds in all the treatment groups were measured and photographs were captured on days 1, 3, 7 and 14. On day 14, all the animals were sacrificed by decapitation, the wounded area was dissected, and a section was stored in the 10% formalin solution for histopathological and immunohistochemical study. The rest parts were flash frozen in liquid nitrogen and then stored at −80 °C for the biochemical analysis.

### 2.11. Wound measurement

The percentage of wound contraction was determined through the following formula:

Wound contraction %=(WD at Day 1−WD at Day 14)/(WD at Day 1)×100

### 2.12. Histopathological analysis

Skin sections fixed with 10% formalin were initially dehydrated and treated with xylene. The sections were dissected and embedded in the paraffin wax and a thin section of 4 µm was cut using a microtome. The cut sections were rehydrated and stained by using hematoxylin-eosin dye and visualized under a light microscope for the possible structural damage in terms of degree/abundance or infiltration of inflammatory cells, the proliferation of fibroblast, deposition of collagen, tissue granulation, re-epithelization and angiogenesis, and scored from − to +++.

### 2.13. Immunohistochemical analysis of TNF-α, IL-6, TGF-β, VEGFA and PDGFR-β

Sections of the tissues were deparaffinized followed by rehydration. Consequently, they were brought to boil for 10 min in citrate buffer (pH 6.0). The blocking solution was in a Cell and Tissue Staining Rabbit Kit and the suitable secondary antibody, and 3,3′-diaminobenzidine (DAB) (Catalog # CTS005 and CTS002, R&D Systems, Minneapolis, MN, USA) was used in conducting this analysis. Incubation of the sections 5% bovine serum albumin was carried out for 2 hrs. Next the sections were kept with the primary antibodies (1 μg/ml); anti-TNF-α (ab220210), anti-IL-6 (ab9324), anti-TGF-β1 (ab215715), anti-VEGFA (ab1316), and anti-PDGFR-β (ab32570) at 4 °C overnight. All primary antibodies were purchased from Abcam, Cambridge, UK. After washing the slides and incubating them for 1 hr in the biotinylated secondary antibody at room temperature. PBS with 0.5% tween 20 was used for washing. This was followed by the application of DAB and observation of color development with a light microscope (Nikon SMZ 1000 with a Nikon DS‐Fi1 digital camera). DPX was used as a mounting solution, where one drop was added to the slides which were then photographed once dry. Analysis of images was carried out with Image J analysis software (ImageJ, 1.46a, NIH, USA).

### 2.14. Biochemical analysis

Skin tissue homogenates (1:10 w/v in phosphate buffer 0.1 M, pH 7.4) were used to assess the biochemical parameters. Concentrations of malondialdehyde (MDA), superoxide dismutase (SOD), and glutathione peroxidase (GPx) concentrations were measured using kits (Biodiagnostic, Cairo, Egypt) according to the manufacturer’s protocol. Total protein and hydroxyproline were measured using Abcam (Cambridge, UK) ELISA kits.

### 2.15. Rt-qPCR for assessing mRNA expression of col 1A1

An ultrasonic probe was used for the homogenization of skin tissues. Extraction of RNA was via a nucleic acid extraction kit (NucleoSpin, Macherey-Nagel GmbH & Co. KG, Duerin, Germany). cDNA was produced with a Reverse Transcription Kit (Applied Biosystems, Foster City, CA, USA). A Taq PCR Master Mix Kit (Qiagen, Valencia, CA, USA) was used for PCR experiments using the primers given in Table 2. Results were expressed in the cycle threshold (Ct). The Ct values of collagen 1A1 (Col1A1) and the housekeeping gene (GAPDH) were included in the PCR datasheet. Relative quantitation (RQ) of Col1A1 depended on the computation of delta-delta Ct (ΔΔCt).

**Table 2.** Primers sequence used for RT-qPCR.

	Forward	Reverse	Gene bank
Col1A1	ATCAGCCCAAACCCCAAGGAGA	CGCAGGAAGGTCAGCTGGATAG	NM_053304.1
GAPDH	CCATTCTTCCACCTTTGATGCT	TGTTGCTGTAGCCATATTCATTGT	NM_017008.4

### 2.16. Statistical analysis

Data were tested for normality using Kolmogorov–Smirnov test. Statistical analysis was done using a one-way analysis of variance followed by Tukey’s post hoc analysis. GraphPad Prism software, version 8.0 (La Jolla, CA, USA) was used to sketch graphs and perform statistical analyses. *P*-values < .05 were taken as significant.

## 3. Results

### 3.1. Selection of optimized GPN-MLT nanoconjugate using experimental design-based analysis

GPN-MLT nanoconjugate formulations were prepared using pH, incubation time, and sonication time as independent factors. At the same time, minimum particle size was selected as the goal of the experimental design for the selection of optimized nanoconjugate. Software yielded fifteen nanoconjugates with the different ratios of independent variables were prepared and obtained observed and fitted particle is shown in Table 3. The observed and fitted values of the design were observed in good agreement.

**Table 3.** Observed and fitted value of particle size obtained from the various runs of experimental design.

Runs	Observed value	Fitted value
1	154.0	165.125
2	421.0	420.125
3	234.0	221.625
4	453.0	441.875
5	187.0	187.875
6	311.0	299.0
7	324.0	323.333
8	321.0	323.333
9	325.0	323.333
10	386.0	398.375
11	298.0	299.25
12	265.0	265.375
13	387.0	399.0
14	398.0	397.625
15	345.0	343.75

The data for analysis of variance for particle size is presented in Table 4. The achieved *p*-value from the experimental design demonstrated the statistically significant effect of selected independent variables on the particle size of various prepared nanoconjugates. Furthermore, a strong link was discovered between pH, incubation time, and sonication time and a remarkable impact on the dependent variable, i.e. particle size. The adjusted *R*^2^ value was 97.5979%, and the *R*^2^ value was 99.1421%.

**Table 4.** Analysis of variance data for particle size of different prepared GPN-MLT nanoconjugate as per the obtained trials from the experimental design.

Source	Sum of squares	Degree of freedom	Mean square	*F*-Ratio	*P*-Value
A: pH	83640.5	1	83640.5	488.89	.0000
B: Incubation time	10440.1	1	10440.1	61.02	.0006
C: Sonication time	1540.13	1	1540.13	9.00	.0301
AA	1072.31	1	1072.31	6.27	.0543
AB	784.0	1	784.0	4.58	.0852
AC	36.0	1	36.0	0.21	.6657
BB	463.853	1	463.853	2.71	.1606
BC	756.25	1	756.25	4.42	.0895
CC	1.85256	1	1.85256	0.01	.9212
Total error	855.417	5	171.083		

The software suggested the polynomial equation for particle size is discussed below as Equation 1. The independent factor regression results indicated that all of the examined factors influenced particle size. In comparison to incubation time (−2.53) and sonication time (−0.34), the regression coefficient for pH (71.84) was recorded highest in the equation. As a result, the independent parameters had the following effect on particle size: pH > incubation time > sonication time.

(1) $$\text{Particle size }=\text{ 17}.0\text{2 }+\text{ 71}.\text{84 A }+\text{ 2}.\text{53 B }-0.\text{34 C }-\text{ 2}.{\text{73 A}}^{\text{2}}-0.\text{28 AB }+0.\text{4}0\text{ AC }+0.0{\text{3 B}}^{\text{2}}-0.\text{23 BC }+0.0{\text{8 C}}^{\text{2}}$$

**Figure 1.**
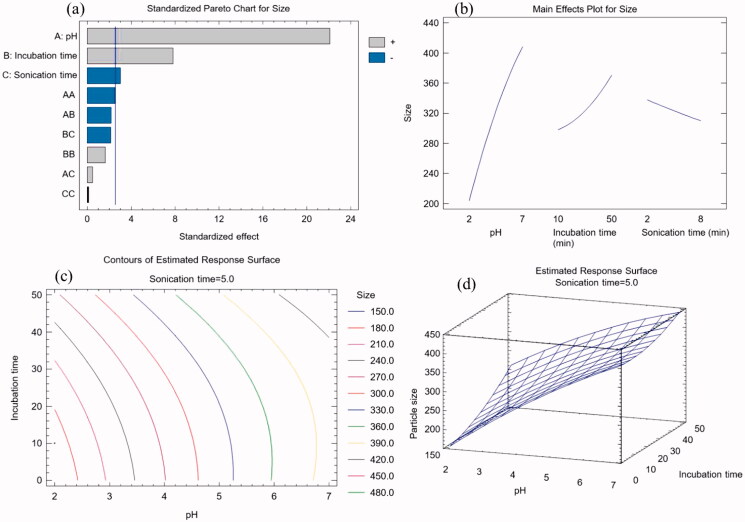
Graphs yielded from experimental design software for the development of GPN-MLT nanoconjugate (a) standard Pareto chart, (b) main effects plot, (c) contour plot, and (d) response surface plot.

The Pareto chart of Figure 1(a) revealed that pH, incubation time, and sonication duration all had substantial effects on particle size. The chart indicated that pH and incubation duration exhibited a positive impact, whereas sonication time had negative effects on the particle size of prepared nanoconjugate. As a result, larger levels of pH and incubation duration increase particle size, but higher levels of sonication duration decrease it. These findings were in line with the conclusions drawn from Equation (1). The main effect graphic also confirmed this tendency (Figure 1b). In the manufacturing process of GPN-MLT nano conjugate, pH and incubation duration have similar to the reaction duration provided time for the indulgence between GB and GL. The conjugation process, i.e. the attachment of the drug, improves as reaction time increases. As a result, increasing the incubation time increases the possibility of additional drug molecules attaching and, as a result, increasing particle size. Meanwhile, increasing the sonication period has been shown to reduce the size of nanoformulations. Thus, the outcomes of the current study's findings can be validated. The contour plot's iso-value curves were more dependent on the pH, matching the findings of the main effects plot (Figure 1c). Increased levels of pH resulted in a considerable elevation of the response surface graph (Figure 1d).

The software suggested the optimum formula conditions for GPN-MLT nanoconjugate for pH, incubation duration, and sonication duration were 2, 10.03 min, and 8.0 min, respectively. Meanwhile, the predicted optimum particle size for GPN-MLT was found to be 162.73 nm and the observed (experimentally carried out) was found to be 156.9 nm.

### 3.2. Effect of GPN-MLT nanoconjugate on wound contraction

**Figure 2.**
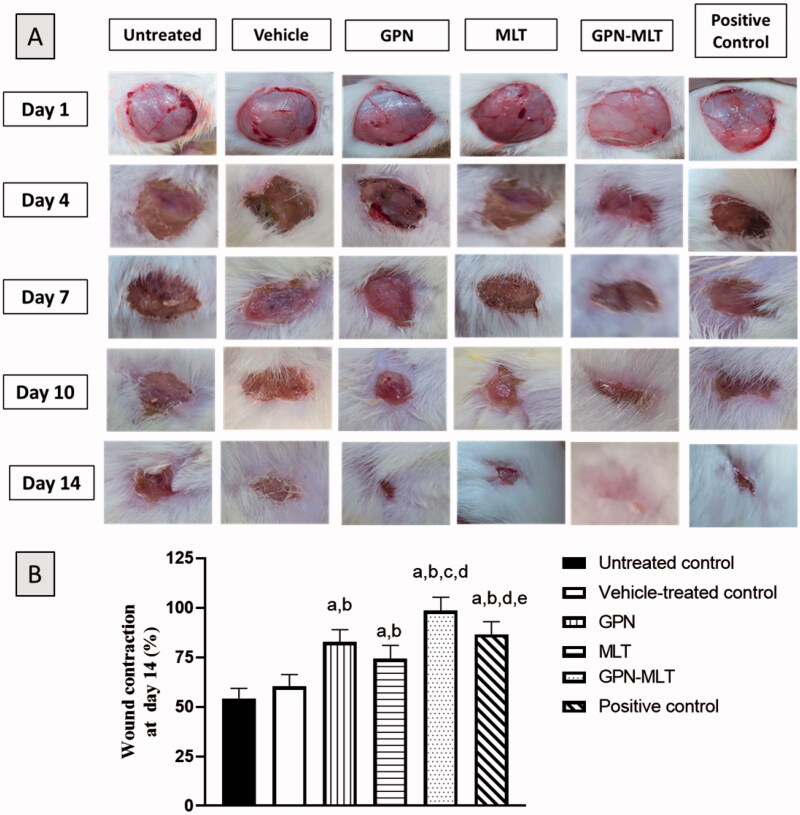
Effects of GPN, MLT, GPN-MLT nanoconjugate and marketed formulation on the wound contraction and % wound contraction on day 14. (a) Significantly different from Untreated control at *p* < .05, (b) Significantly different from Vehicle-treated control at *p* < .05, (c) Significantly different from GPN at *p* < .05, (d) Significantly different from MLT at *p* < .05, (e) Significantly different from GPN-MLT at *p* < .05.

As depicted in Figure 2, untreated group and vehicle-treated group, conventional formulation of GPN, MLT, nanoconjugate of GPN-MLT and positive control formulation had open wounds on day 1. However, on days 4, 7, 10 and 14, the topical application of nanoconjugate of GPN-MLT exhibited a significant increase in wound contraction and closure of wound followed by the marketed formulation, GPN alone and MLT alone.

### 3.3. Effect of GPN-MLT nanoconjugate on histological changes of wounded skin (H & E and MT staining)

**Figure 3.**
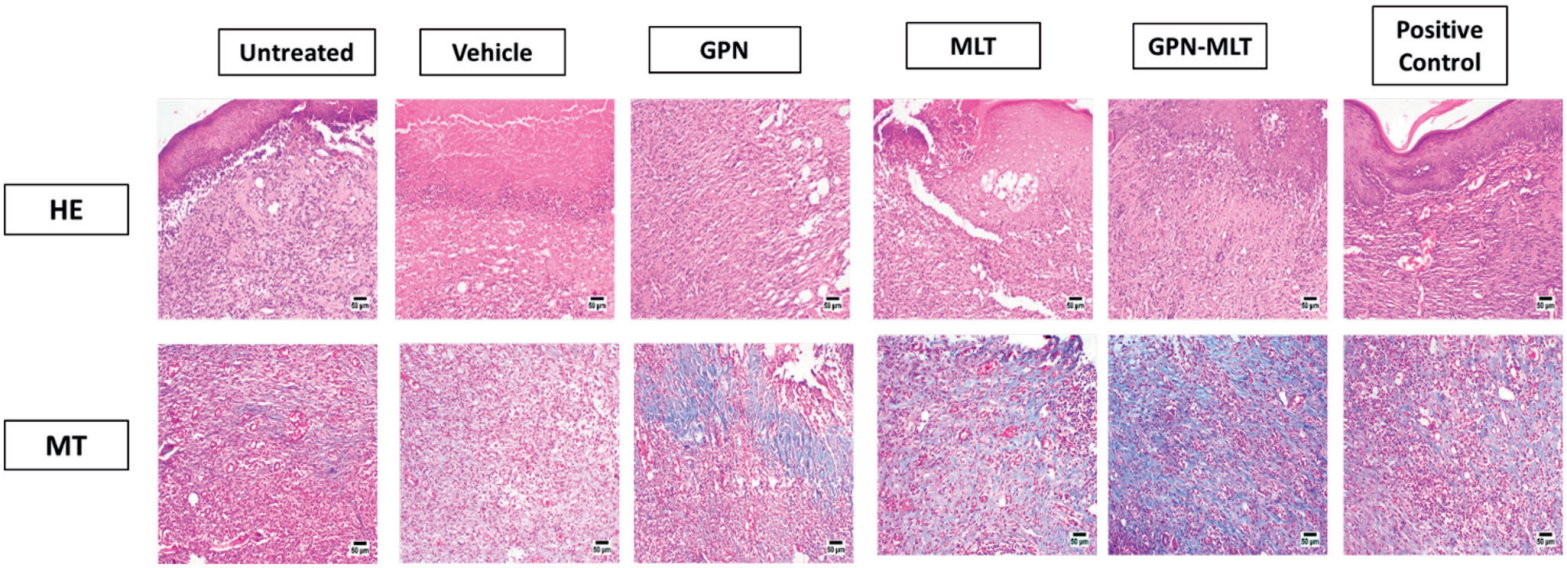
Effects of GPN, MLT, GPN-MLT nanoconjugate and marketed formulation on the histopathological analysis (A) H & E staining and (B) MT staining.

As shown in Figure 3, the epidermal layer of the untreated group and vehicle-treated showed marked focal hyperkeratosis, and acanthosis, whereas the dermal layer showed an increased deposition of collagen fibers along with the scattered as well as proliferating blood vessels and an extensive chronic infiltrate of inflammatory cells. These are the distinct features of intact chronic wounds. When the animals were treated with the conventional formulation of GPN, MLT, nanoconjugate of GPN-MLT, and the marketed formulation, a marked reduction in the histopathological damage was seen, whereas the conventional formulations and marketed formulation exhibited almost similar effects as shown in Figure 3 and Table 5.

**Table 5.** Histological features of wound healing in animals treated with GPN, MLT, or GPN-MLT on day 14.

	Untreated control	Vehicle	GPN	MLT	GPN-MLT	Positive control
RE	−	**−/+**	++	**++**	++	++
FP	++	++	+	+	+	+
CD	++	+	+	++	+++	++
IC	++	++	+	+	+/**−**	+
Phase I	++	++	+	+	+	+
Phase II	++	++	+++	++	+++	++
Phase III	**−**	**−**	++	++	+++	++

RE = re-epithelization, FP = fibroblast proliferation, CD = collagen deposition, IC = inflammatory cell infiltration, Phase I = inflammation phase of wound healing, Phase II = proliferation phase of wound healing, Phase III = re-modeling phase of wound healing.

MT staining was also performed to estimate the pro-fibrotic effect of different formulations. It was found that untreated and vehicle-treated groups showed a marked reduction in fibrosis as evident from the appearance of blue color (presence of collagens). When the animals were treated with the conventional formulation of GPN, MLT, nanoconjugate of GPN-MLT and the marketed formulation, marked elevation in the fibrotic tissue was seen in the GPN-MLT nanoconjugate, followed by the marketed formulations, MLT and GPN.

### 3.4. Effect of GPN-MLT nanoconjugate on antioxidant status

**Figure 4.**
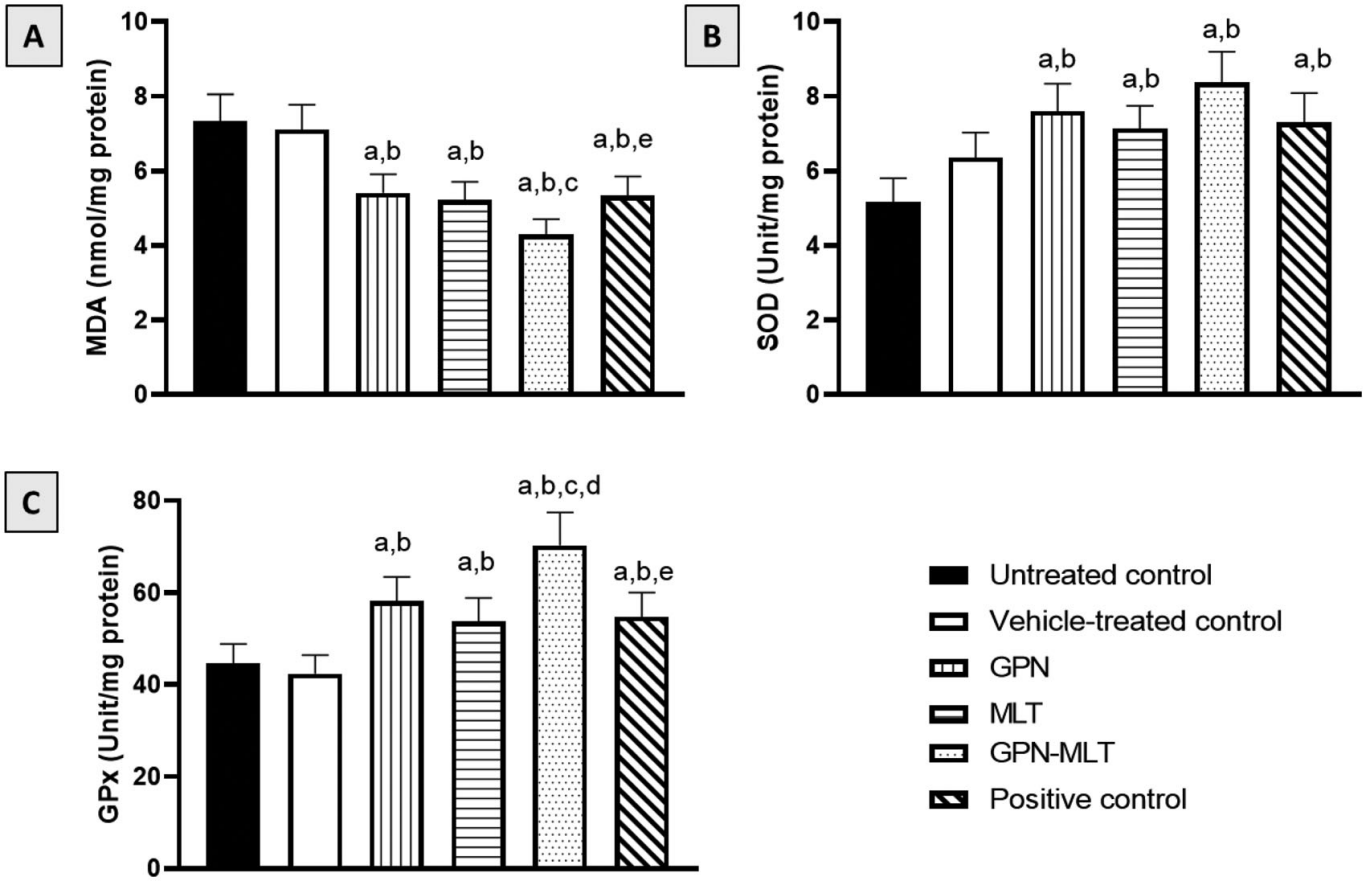
Effects of GPN, MLT, GPN-MLT nanocomplex and marketed formulation on the markers of antioxidant enzymes (A) MDA, (B) SOD, and (C) GPx. a: Significantly different from Untreated control at *p* < .05, b: Significantly different from Vehicle-treated control at *p* < .05, c: Significantly different from GPN at *p* < .05, d: Significantly different from MLT at *p* < .05, e: Significantly different from GPN-MLT at *p* < .05.

In the present study, when the markers of oxidative stress such as MDA concentration and SOD and GPx activities were estimated, the untreated group showed a significant accumulation of MDA (Figure 4A) accompanied by a reduction in SOD and GPx activities (Figure 4B,C). When the animals were treated with the conventional formulation of GPN, MLT, nanoconjugate of GPN-MLT and marketed formulation, significant increment in the activity of SOD and GPx and reduction in the level of MDA were found in the GPN-MLT nanoconjugate group as compared to the conventional and marketed formulations.

### 3.5. Effect of GPN-MLT nanoconjugate on inflammatory markers

**Figure 5.**
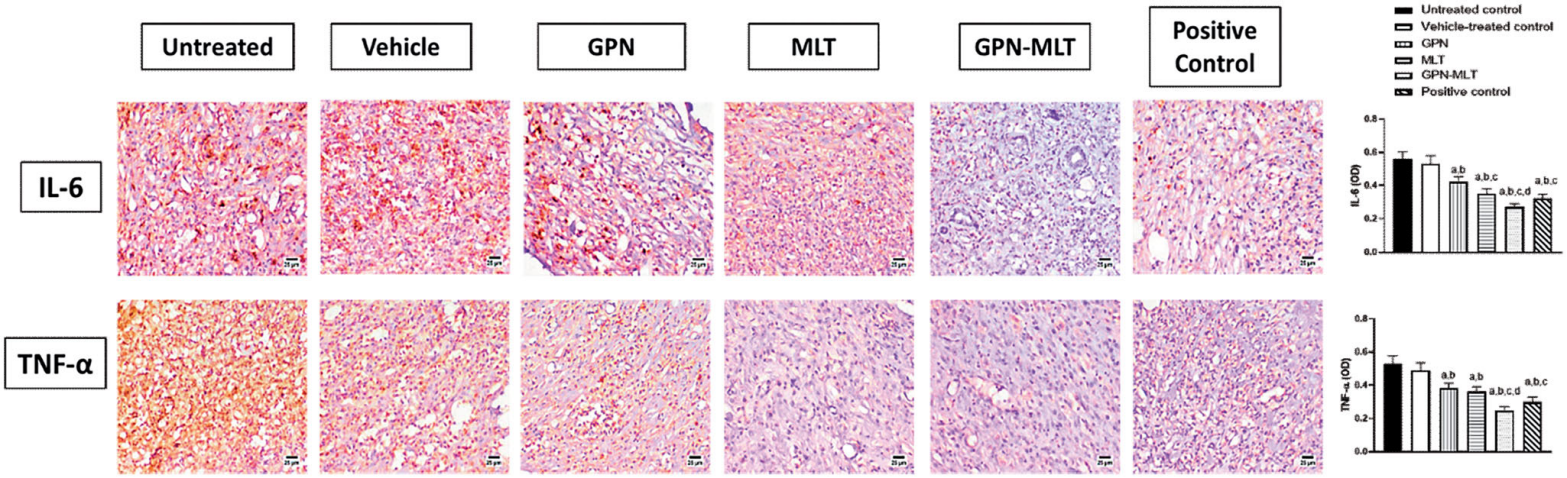
Effects of GPN, MLT, GPN-MLT nanocomplex and marketed formulation on the expression level of (A) IL-6, and (B) TNF-α. a: Significantly different from Untreated control at *p* < .05, b: Significantly different from Vehicle-treated control at *p* < .05, c: Significantly different from GPN at *p* < .05, d: Significantly different from MLT at *p* < .05.

In the present study, when the markers of inflammation such as IL-6 and TNF-α were estimated, the untreated group showed significant elevation in their level. When the animals were treated with the conventional formulation of GPN, MLT, nanoconjugate of GPN-MLT and marketed formulation, a significant reduction in the expression level of IL-6 and TNF-α was found in the GPN-MLT nanoconjugate group, followed by the marketed formulation, MLT and GPN alone as shown in Figure 5.

### 3.6. Effect of GON-MLT nanoconjugate accelerate on fibrosis and angiogenesis markers

**Figure 6.**
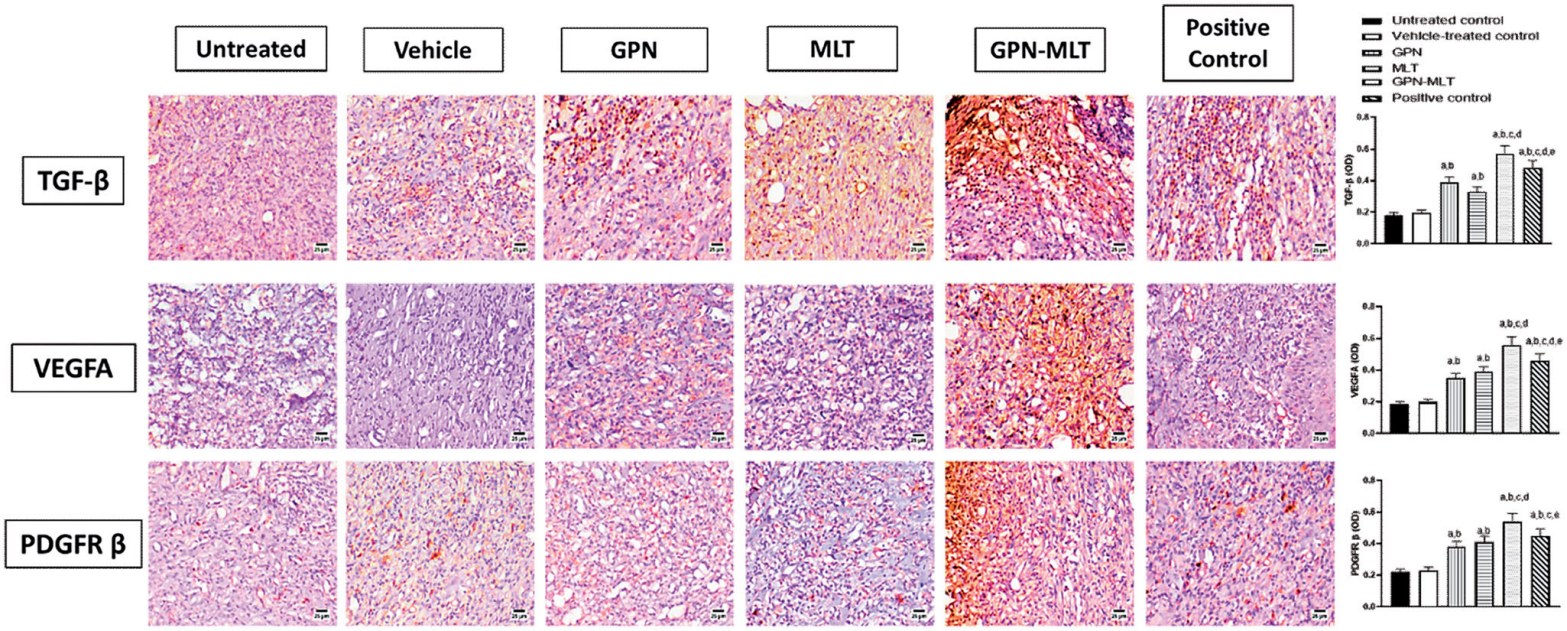
Effects of GPN, MLT, GPN-MLT nanoconjugate, and marketed formulation on the expression level of (A) TGF-β (B) VEGFA and (C) PDGFR-β. a: Significantly different from Untreated control at *p* < .05, b: Significantly different from Vehicle-treated control at *p* < .05, c: Significantly different from GPN at *p* < .05, d: Significantly different from MLT at *p* < .05, e: Significantly different from GPN-MLT at *p* < .05.

In the present study, when the markers of fibrosis and angiogenesis, such as TGF-β, VEGFA, and PDGFR-β, were estimated, the untreated group showed a significant reduction in their level. When the animals were treated with the conventional formulation of GPN, MLT, nanoconjugate of GPN-MLT and marketed formulation, significant elevation in the expression level of TGF-β, VEGFA, and PDGFR-β was found in the GPN-MLT nanoconjugate group, followed by the marketed formulation, MLT and GPN alone for VEGFA and PDGFR-β where in case of TGF-β, GPN alone exhibited superiority as compared to the MLT alone shown in Figure 6.

### 3.7. Effect of GPN-MLT nanoconjugate on markers of collagen deposition

**Figure 7.**
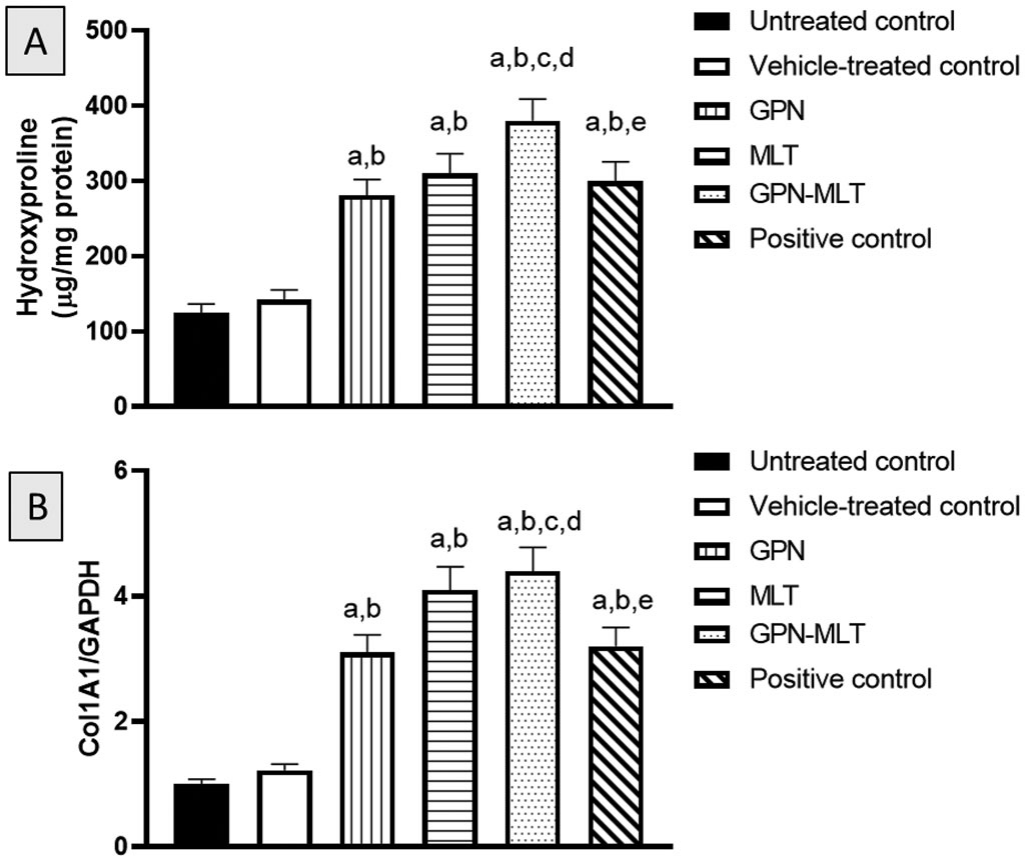
Effects of GPN, MLT, GPN-MLT nanoconjugate and marketed formulation on the level of (A) hydroxyproline and (B) Col1A1. a: Significantly different from Untreated control at *p* < .05, b: Significantly different from Vehicle-treated control at *p* < .05, c: Significantly different from GPN at *p* < .05, d: Significantly different from MLT at *p* < .05, e: Significantly different from GPN-MLT at *p* < .05.

In the present study, when the markers of collagen deposition such as hydroxyproline content and mRNA expression of Col1A1 were estimated, the untreated group showed a significant reduction in their level. When the animals were treated with the conventional formulation of GPN, MLT, nanoconjugate of GPN-MLT and marketed formulation, significant elevation in the expression level of hydroxyproline and Col1A1 was found in the GPN-MLT nanoconjugate group, followed by the MLT alone, marketed formulation, and GPN alone as shown in Figure 7(A,B).

## 4. Discussion

The process of wound healing in diabetes is slow and inefficient management can bring about complications (Sharp & Clark, 2011). The current study involved optimization of a new GPN-MLT nanoconjugate formulation (Table 4 and Figure 1). The wound healing features of the optimized formula were evaluated in streptozotocin-induced diabetic rats as well as the potential synergistic interaction between these two compounds (Figure 2). In the field of drug delivery, nanoparticulate systems with a size < 400 nm have recently gained attention (Yingchoncharoen et al., 2016). Tissue penetration could be improved by lowering the size to the smallest achievable value to increase the surface area accessible for tissue penetration (Badr-Eldin et al., 2021). Therefore, the study focused on reducing the size of the nanospheres. The particle size of the optimized GPN-MLT nanoconjugate was 156.9 nm.

Our data indicated that GNP-MLT nanoconjugate offered a significantly expedited wound healing activity. This was confirmed by histological examinations which indicated that the nano conjugate-treated wounds showed almost complete healing as they reached the final phases of healing with re-modelling and collagen deposition (Figure 3 and Table 5). These findings gain support from the previously reported ability of GPN to enhance wound healing in rats (Sarıtaş et al., 2016). Further, MLT nanoconjugates considerably expedited the healing of wounds in normoglycemic and hyperglycemic rats (Alhakamy et al., 2021b; Eid et al., 2022). In addition, MLT positive effects are consistent with the documented wound healing activities of bee venom. It has been reported that it brings about an accelerated curative effect and could be applied as a new potential treatment for wound repair (Kurek-Górecka et al., 2020).

The mechanisms of impaired healing response are not fully characterized. However, accumulating evidence indicates a role of oxidative stress in the pathogenesis of delayed healing of wounds (Schäfer & Werner, 2008). This is of particular significance in diabetes (Deng et al., 2021) to the extent of recommending the use of antioxidants to treat diabetic wounds (Zhang et al., 2021). Therefore, we have explored the potential of optimized GPN-MLT formula to prevent oxidative stress induced by wounding. Fortunately, the nanoconjugate formula showed significant antioxidant properties as evidenced by inhibition of lipid peroxidation and antioxidant enzyme exhaustion (Figure 4). Our data are consistent with the reported antioxidant activities of GPN compounds (Saleem et al., 2021). Also, GPN antioxidant properties offered neuroprotective actions in a rat model of cerebral ischemia-reperfusion injury (Zhang et al., 2021). In addition, MLT antioxidant properties have been previously reported in an experimental model of lung injury (El-Aarag et al., 2019). Moreover, MLT antioxidant activity in healing wounds has been shown in rats (Alhakamy et al., 2021a, 2021b). Thus, it can be suggested that the enhanced antioxidative properties of the optimized formula participate in the observed expedition of wound healing in diabetic rats.

Inflammation is considered a nonspecific immune reaction, involving the degeneration of tissue, that usually resolves after infiltrated leukocytes revert to their pre-inflammatory condition. A chemotactic response in leukocytes by inflammatory cytokines enhances the inflammation phase (Henry & Garner, 2003). Therefore, inflammation represents the first phase of wound healing. Our data indicate that treatment GPN–MLT significantly expedited the inflammatory phase as indicated by the reduced expression of IL-6, and TNF-α (Figure 5). Experimentally, GPN anti-inflammatory activity has been previously shown (Abdel-Salam & El-Batran, 2005; Motavallian et al., 2021). On the other side, MLT anti-inflammatory properties have been reported (Park et al., 2007; Kim et al., 2018; Motavallian et al., 2021). In particular, its anti-inflammatory actions in wounded skin have been proven in rats (Alhakamy et al., 2021a, 2021b). MLT antioxidant and anti-inflammatory have been reported to involve modulation of TLR4/TRAF6 mediated NF-κB and p38MAPK pathways (Ahmedy et al., 2020). Similarly, the anti-inflammatory activity of GPN has been attributed to the modulation of NF-κB (Li et al., 2022b). These data give a base for explaining the observed enhancement of the anti-inflammatory effects of the nanoconjugate formula.

Late phases of wound healing involve and remodeling, where the growth and angiogenic factors and collagen deposition play a decisive role (Martin & Nunan, 2015). In this regard, TGF- β family has been reported to play a crucial role in wound healing as it regulated fibrosis, inflammation and angiogenesis (Ferguson & O’Kane, 2004). Our data indicated that both GPN and MLT enhance TGF-β1 expression. This gains indirect support from the ability of GPN to up-regulate the expression of genes encoding for TGF-β in rat cultured dorsal root ganglion (Heo et al., 2013). Also, our results indicated that MLT enhances the expression of TGF-β1 which coincides with previous data highlighting its ability to promote wound healing in a pathway involving TGF-β1 (Eid et al., 2022). Further, MLT exhibited pro-angiogenic properties in healing wounds as evidenced by increased expression of VEGFA and PDGFR (Figure 6). This is in line with the known role of angiogenesis and angiogenic factors in wound healing (Bao et al., 2009). It has been reported that PDGFR-β activation is essential for fibroblast recruitment and function in wound healing (Rajkumar et al., 2006). In general bee venom has been reported to enhance angiogenesis via increasing VEGF expression (Kurek-Górecka et al., 2020). Remodeling is the last but critical stage of wound healing in which tissue integrity is restored. It is characterized by the generation of new epithelial cells and scar formation (Sorg et al., 2017). In this regard, collagen and its derived peptides are highly involved and have been reported to exhibit advantageous effects in wound healing (Yao et al., 2008). In the current study, hydroxyproline content and Col 1A1 mRNA expression were enhanced by GPN and MLT (Figure 7). This is consistency with our results that showed their positive effects on TGF-β1 expression and consequently collagen deposition (Carpena et al., 2020). Our observations are also strengthened by the ability of MLT to enhance hydroxyproline concentration and mRNA expression of Col1A1 in healing skin of wounded rats (Kurek-Górecka et al., 2020). Collagen type I is the most common type of collagens in healing skin tissues (Mathew-Steiner et al., 2021). In particular, Col 1A1 has been considered a reliable marker of collagen synthesis in the late stages of wound healing (Meephansan et al., 2017). In most of the assessed parameters including wound contraction, the formulated nanoconjugate exhibited superior healing activities.

## Conclusion

The present study highlights the potential of a novel GPN-MLT nanoconjugate to accelerate wound healing in diabetic rats. The optimized nanoconjugate, with respect to particle size, possessed greater wound healing properties compared to individual components, following 14 days of daily topical application. This effect could be attributed to its ability to counteract oxidative stress and expedite inflammatory phases. The GPN-MLT nanoconjugate was also found to process angiogenic and pro-collagen activities that have an important role in wound healing. Overall, these findings present a novel optimized formula that could accelerate wound healing in diabetes.

## References

[CIT0001] Abdel-Salam OME, El-Batran S. (2005). Pharmacological investigation of trimetazidine in models of inflammation, pain and gastric injury in rodents. Pharmacology 75:122–32. 16155371 10.1159/000088211

[CIT0002] Al-Ani I, Zimmermann S, Reichling J, Wink M. (2015). Pharmacological synergism of bee venom and melittin with antibiotics and plant secondary metabolites against multi-drug resistant microbial pathogens. Phytomedicine 22:245–55. 25765829 10.1016/j.phymed.2014.11.019

[CIT0003] Alhakamy NA, Ahmed OAA, Fahmy UA, Shadab M. (2021a). Apamin-conjugated alendronate sodium nanocomplex for management of pancreatic cancer. Pharmaceuticals 14:729. 34451826 10.3390/ph14080729 PMC8398389

[CIT0004] Alhakamy NA, Caruso G, Eid BG, et al. (2021b). Ceftriaxone and melittin synergistically promote wound healing in diabetic rats. Pharmaceutics 13:1622. 34683915 10.3390/pharmaceutics13101622 PMC8539663

[CIT0005] Ahmedy OA, Ibrahim SM, Salem HH, Kandil EA. (2020). Antiulcerogenic effect of melittin via mitigating TLR4/TRAF6 mediated NF-ΚB and p38MAPK pathways in acetic acid-induced ulcerative colitis in mice. Chem Biol Interact 331:109276. 33002459 10.1016/j.cbi.2020.109276

[CIT0006] Badr-Eldin SM, Aldawsari HM, Ahmed OA, et al. (2021). Optimized semisolid self-nanoemulsifying system based on glyceryl behenate: a potential nanoplatform for enhancing antitumor activity of raloxifene hydrochloride in MCF-7 human breast cancer cells. Int J Pharm 600:120493. 33744452 10.1016/j.ijpharm.2021.120493

[CIT0007] Bao P, Kodra A, Tomic-Canic M, et al. (2009). The role of vascular endothelial growth factor in wound healing. J Surg Res 153:347–58. 19027922 10.1016/j.jss.2008.04.023 PMC2728016

[CIT0008] Carpena M, Nuñez-Estevez B, Soria-Lopez A, Simal-Gandara J. (2020). Bee venom: an updating review of its bioactive molecules and its health applications. Nutrients 12:3360–27. 10.3390/nu12113360 PMC7693387 33142794

[CIT0009] Deng L, Du C, Song P, et al. (2021). The role of oxidative stress and antioxidants in diabetic wound healing. Oxid Med Cell Longev 2021:8852759. 33628388 10.1155/2021/8852759 PMC7884160

[CIT0010] Eid BG, Alhakamy NA, Fahmy UA, et al. (2022). Melittin and diclofenac synergistically promote wound healing in a pathway involving TGF-β1. Pharmacol Res 175:105993. 34801680 10.1016/j.phrs.2021.105993

[CIT0011] El-Aarag B, Magdy M, AlAjmi MF, et al. (2019). Melittin exerts beneficial effects on paraquat-induced lung injuries in mice by modifying oxidative stress and apoptosis. Molecules 24:1–8. 10.3390/molecules24081498 PMC6514788 30995821

[CIT0012] Ferguson MWJ, O'Kane S. (2004). Scar-free healing: from embryonic mechanisms to adult therapeutic intervention. Philos Trans R Soc Lond B Biol Sci 359:839–50. 15293811 10.1098/rstb.2004.1475 PMC1693363

[CIT0013] Gajski G, Garaj-Vrhovac V. (2013). Melittin: a lytic peptide with anticancer properties. Environ. Toxicol. Pharmacol 697–705. 10.1016/j.etap.2013.06.009 23892471

[CIT0014] Gudeman J, Jozwiakowski M, Chollet J, Randell M. (2013). Potential risks of pharmacy compounding. Drugs R D 13:1–8. 23526368 10.1007/s40268-013-0005-9 PMC3627035

[CIT0015] Henry G, Garner WL. (2003). Inflammatory mediators in wound healing. Surg Clin North Am 83:483–507. 12822721 10.1016/S0039-6109(02)00200-1

[CIT0016] Heo JH, Lee SH, Chang KH, et al. (2013). Identification of differentially expressed genes by gabapentin in cultured dorsal root ganglion in a rat neuropathic pain model. Biomol Ther 21:126–31. 10.4062/biomolther.2013.014 PMC3762310 24009870

[CIT0017] Jeong YJ, Shin JM, Bae YS, et al. (2015). Melittin has a chondroprotective effect by inhibiting MMP-1 and MMP-8 expressions via blocking NF-κB and AP-1 signaling pathway in chondrocytes. Int Immunopharmacol 25:400–5. 25708656 10.1016/j.intimp.2015.02.021

[CIT0018] Jiang L, Xiong Y, Cui J. (2022). Comparison of the efficacy and safety of duloxetine and gabapentin in diabetic peripheral neuropathic pain: a meta-analysis. Contrast Media Mol Imaging 2022:4084420. 35299589 10.1155/2022/4084420 PMC8904906

[CIT0019] Kim WH, An HJ, Kim JY, et al. (2018). Anti-inflammatory effect of melittin on porphyromonas gingivalis LPS-stimulated human keratinocytes. Molecules 23:332. 10.3390/molecules23020332 PMC6017529 29401750

[CIT0020] Kukkar A, Bali A, Singh N, Jaggi AS. (2013). Implications and mechanism of action of gabapentin in neuropathic pain. Arch Pharm Res 36:237–51. 23435945 10.1007/s12272-013-0057-y

[CIT0021] Kurek-Górecka A, Komosinska-Vassev K, Rzepecka-Stojko A, Olczyk P. (2020). Bee venom in wound healing. Molecules 26:148–13. 10.3390/molecules26010148 PMC7795515 33396220

[CIT0022] Lee G, Bae H. (2016). Anti-inflammatory applications of melittin, a major component of bee venom: detailed mechanism of action and adverse effects. Molecules 21:616. 10.3390/molecules21050616 PMC6273919 27187328

[CIT0023] Li T, Sun M, Wu S. (2022a). State-of-the-art review of electrospun gelatin-based nanofiber dressings for wound healing applications. Nanomater 12:784. 10.3390/nano12050784 PMC8911957 35269272

[CIT0024] Li J, Xu K, Ding H, Xi Q. (2022b). Gabapentin reduces alcohol intake in rats by regulating NF-κB signaling pathway via PPAR γ. Alcohol Alcohol 57:234–41. 34553211 10.1093/alcalc/agab065

[CIT0025] Martin P, Nunan R. (2015). Cellular and molecular mechanisms of repair in acute and chronic wound healing. Br J Dermatol 173:370–8. 26175283 10.1111/bjd.13954 PMC4671308

[CIT0026] Mathew-Steiner SS, Roy S, Sen CK. (2021). Collagen in wound healing. Bioengineering 8:63. 34064689 10.3390/bioengineering8050063 PMC8151502

[CIT0027] Meephansan J, Rungjang A, Yingmema W, et al. (2017). Effect of astaxanthin on cutaneous wound healing. Clin Cosmet Investig Dermatol 10:259–65. 10.2147/CCID.S142795 PMC5516620 28761364

[CIT0028] Memariani H, Memariani M. (2020). Anti-fungal properties and mechanisms of melittin. Appl Microbiol Biotechnol 104:6513–26. 32500268 10.1007/s00253-020-10701-0

[CIT0029] Motavallian A, Bouzari S, Zamani E, et al. (2021). An investigation of the anti-inflammatory effects of gabapentin on acetic acid-induced colitis in rats. Mol Biol Rep 48:3423–30. 33928442 10.1007/s11033-021-06357-2

[CIT0030] Ola MS, Alhomida AS, LaNoue KF. (2019). Gabapentin attenuates oxidative stress and apoptosis in the diabetic rat retina. Neurotox Res 36:81–90. 30830678 10.1007/s12640-019-00018-w

[CIT0031] Park HJ, Son DJ, Lee CW, et al. (2007). Melittin inhibits inflammatory target gene expression and mediator generation via interaction with IkappaB kinase. Biochem Pharmacol 73:237–47. 17067557 10.1016/j.bcp.2006.09.023

[CIT0032] Patel S, Srivastava S, Singh MR, Singh D. (2019). Mechanistic insight into diabetic wounds: pathogenesis, molecular targets and treatment strategies to pace wound healing. Biomed Pharmacother 112:108615. 30784919 10.1016/j.biopha.2019.108615

[CIT0033] Qing C. (2017). The molecular biology in wound healing & non-healing wound. Chin J Traumatol 20:189–93. 28712679 10.1016/j.cjtee.2017.06.001 PMC5555286

[CIT0034] Rajkumar VS, Shiwen X, Bostrom M, et al. (2006). Platelet-derived growth factor-beta receptor activation is essential for fibroblast and pericyte recruitment during cutaneous wound healing. Am J Pathol 169:2254–65. 17148686 10.2353/ajpath.2006.060196 PMC1762470

[CIT0035] Rose MA, Kam PCA. (2002). Gabapentin: pharmacology and its use in pain management. Anaesthesia 57:451–62. 11966555 10.1046/j.0003-2409.2001.02399.x

[CIT0036] Roy R, Zayas J, Singh SK, et al. (2022). Overriding impaired FPR chemotaxis signaling in diabetic neutrophil stimulates infection control in murine diabetic wound. Elife 11:e72071. 10.7554/eLife.72071 PMC8846594 35112667

[CIT0037] Saleem MF, Khan MA, Ahmad I, et al. (2021). Synthesis and characterization of some new Schiff base derivatives of gabapentin, and assessment of their antibacterial, antioxidant and anticonvulsant activities. Trop J Pharm Res 20:145–53.

[CIT0038] Sarıtaş TB, Korkmaz M, Sevimli A, Sarıtaş ZK. (2016). Comparison of the effects of gabapentin and pregabalin on wound healing in rats. Int Wound J 13:748–53. 25348659 10.1111/iwj.12364 PMC7949902

[CIT0039] Schäfer M, Werner S. (2008). Oxidative stress in normal and impaired wound repair. Pharmacol Res 58:165–71. 18617006 10.1016/j.phrs.2008.06.004

[CIT0040] Sharp A, Clark J. (2011). Diabetes and its effects on wound healing. Nurs Stand 25:41–7. 10.7748/ns2011.07.25.45.41.c8626 21850847

[CIT0041] Siddiqui A, Alayoubi A, El-Malah Y, Nazzal S. (2014). Modeling the effect of sonication parameters on size and dispersion temperature of solid lipid nanoparticles (SLNs) by response surface methodology (RSM). Pharm Dev Technol 19:342–6. 23590412 10.3109/10837450.2013.784336

[CIT0042] Sorg H, Tilkorn DJ, Hager S, et al. (2017). Skin wound healing: an update on the current knowledge and concepts. Eur Surg Res 58:81–94. 27974711 10.1159/000454919

[CIT0043] Strecker-McGraw MK, Jones TR, Baer DG. (2007). Soft tissue wounds and principles of healing. Emerg Med Clin North Am 25:1–22. 17400070 10.1016/j.emc.2006.12.002

[CIT0044] Tottoli EM, Dorati R, Genta I, et al. (2020). Skin wound healing process and new emerging technologies for skin wound care and regeneration. Pharmaceutics 12:735–0. 10.3390/pharmaceutics12080735 PMC7463929 32764269

[CIT0045] Velnar T, Bailey T, Smrkolj V. (2009). The wound healing process: an overview of the cellular and molecular mechanisms. J Int Med Res 37:1528–42. 19930861 10.1177/147323000903700531

[CIT0046] Wan R, Weissman JP, Grundman K, et al. (2021). Diabetic wound healing: the impact of diabetes on myofibroblast activity and its potential therapeutic treatments. Wound Repair Regen 29:573–81. 34157786 10.1111/wrr.12954

[CIT0047] Yao C, Markowicz M, Pallua N, et al. (2008). The effect of cross-linking of collagen matrices on their angiogenic capability. Biomaterials 29:66–74. 17935778 10.1016/j.biomaterials.2007.08.049

[CIT0048] Yingchoncharoen P, Kalinowski DS, Richardson DR. (2016). Lipid-based drug delivery systems in cancer therapy: what is available and what is yet to come. Pharmacol Rev 68:701–87. 27363439 10.1124/pr.115.012070 PMC4931871

[CIT0049] Zhang W, Chen L, Xiong Y, et al. (2021). Antioxidant therapy and antioxidant-related bionanomaterials in diabetic wound healing. Front Bioeng Biotechnol 9:707479. 34249895 10.3389/fbioe.2021.707479 PMC8264455

